# A Qualitative Study Exploring the Mechanisms Underlying Self‐Management Behaviours Among Community‐Dwelling Type 2 Diabetes Mellitus Patients in Nanjing, China

**DOI:** 10.1111/hex.70522

**Published:** 2025-12-08

**Authors:** Jinxin Li, Yue Zhang, Nan Zhao, Jianxiao Zheng, Yang Wang, Jiajia Dong, Jianwen Zhu, Jie Fu, Hong Fan

**Affiliations:** ^1^ School of Nursing Nanjing Medical University Nanjing China; ^2^ School of Public Health Nanjing Medical University Nanjing China

**Keywords:** community, mechanisms of influence, self‐management behaviour, theory of planned behaviour, type 2 diabetes

## Abstract

**Background:**

Effective self‐management is essential for improving the health outcomes of patients with type 2 diabetes mellitus (T2DM). However, many patients fail to achieve ideal self‐management. This qualitative study, grounded in the theory of planned behaviour (TPB), aims to explore the mechanisms underlying self‐management behaviours of patients with T2DM in the community, to provide insights for improve patients' self‐management and reduce the disease burden.

**Methods:**

Within the framework of the TPB, a semi‐structured interview guide was developed based on a literature review and expert consensus. Qualitative data were obtained from in‐depth interviews with 21 patients with T2DM from a community health service centre in Nanjing, Jiangsu Province. All interviews were conducted by the same interviewer to ensure consistency. Content analysis was used to identify the key factors influencing patients' self‐management behaviours and the underlying mechanism.

**Results:**

This study systematically explained the underlying mechanisms of self‐management behaviours among patients with T2DM based on Ajzen's TPB, including three key levels: (1) Attitudes towards self‐management: shaped by both internal (e.g., self‐perception, perceived importance and understanding of the disease) and external factors (e.g., perceived benefits and degree of acceptance). (2) Subjective norms: influenced by both intrinsic (e.g., personal motivations) and extrinsic pressures (e.g., surrounding individuals and social groups). (3) Perceived behavioural control: determined by both inner (e.g., personal characteristics, physical and mental experience, economic resources and time management) and outer factors (e.g., environmental suitability, policy support and technical availability).

**Conclusion:**

This study provides a valuable theoretical basis and practical implications for designing effective interventions to improve self‐management behaviours of patients with T2DM in the community. To optimize self‐management behaviours of this population, a multifaceted approach is recommended, focusing on the following six key areas: improving T2DM knowledge, strengthening follow‐up management system, empowering patients to take an active role in their care, fostering social support network, developing their own coping skills and expanding policy support for T2DM care.

**Patient or Public Contribution:**

Patients with T2DM were actively involved in this study, providing essential insights and feedback throughout. Their first‐hand experiences contributed to the identification of sub‐categories and categories. Participants also reviewed and confirmed the accuracy of the interview transcripts, ensuring the reliability of the data.

**Abbreviations**
DM: diabetes mellitus
DSM: diabetes self‐management
T2DM: type 2 diabetes mellitus
TPB: theory of planned behaviour

## 1 Introduction

Diabetes mellitus (DM) is a chronic lifelong metabolic disease characterized by hyperglycaemia [1]. With increasing prevalence, DM poses a significant threat to public health and healthcare systems worldwide. Uncontrolled hyperglycaemia can cause long‐term and irreversible damage to health [2], affecting the quality of life of patients and imposing a significant economic burden on families and societies. In 2022, an estimated 828 million adults worldwide were living with diabetes, a substantial increase from 630 million in 1990 [3]. China is among the countries most affected, with 148 million individuals living with diabetes in 2022, second only to India globally [3]. According to reports, global health expenditure related to diabetes was estimated at $966 billion in 2021 and is projected to reach $1.054 trillion by 2045, marking a 9.1% increase from 2021 [4]. Type 2 diabetes mellitus (T2DM) accounts for over 95% of all diabetes cases [5]. With the acceleration of population ageing and urbanization, the economic burden of diabetes in China continues to increase globally [6]. It is predicted that the healthcare expenditure related to diabetes in China will increase from $250.2 billion to $460.4 billion between 2020 and 2030 [6].

T2DM cannot be completely cured at present, but it can be effectively controlled through multifaceted comprehensive treatment and continuous self‐management by patients [7]. The 20th National Congress of the Communist Party proposed strengthening the management of major chronic diseases and improving grassroots healthcare capabilities [8]. Diabetes self‐management (DSM) refers to all activities undertaken by patients to care for their disease, promote health and prevent the long‐term and short‐term effects of diabetes [9]. In 2007, the American Association of Diabetes Educators (AADE) established seven behavioural standards for DSM, primarily including healthy eating, regular exercise, self‐monitoring, medication adherence, problem‐solving, healthy coping and risk factor reduction [10]. DSM aims to control blood glucose and reduce the risk of diabetes‐related complications [11]. However, many patients with T2DM struggle with recommended practices [12]. A survey conducted by Ji et al. on 435 patients with T2DM showed that only 9.2% of them scored well in self‐management behaviours [13]. Studies have shown that the self‐management behaviours of patients with T2DM in China are not optimistic and are at a moderate or low level [14, 15, 16]. Patients with T2DM generally scored higher in dietary control and medication compliance, and lower in exercise, blood glucose monitoring and foot care [17, 18, 19]. The study found that about half of patients with T2DM in China monitor their blood glucose only once a month, and only 7.02% of patients meet the requirements [20]. Moreover, with the extension of disease duration, the dietary control, blood glucose monitoring and medication adherence of patients with T2DM may decline over time [19, 21]. Therefore, there is an urgent need to unearth the deep‐rooted causes that influence patients' self‐management behaviours.

Currently, several quantitative cross‐sectional studies have been conducted in China on the factors influencing self‐management behaviours among community‐dwelling patients with T2DM [22, 23, 24]. However, the underlying mechanisms of these behaviours still require deep exploration. Understanding the processes driving behaviours is crucial for effective interventions, particularly within the complex interplay of cultural, gender, environmental and socioeconomic factors. Building upon previous quantitative research on the influencing factors of self‐management behaviours among community‐dwelling patients with T2DM, [25] this study employed a qualitative approach grounded in the theory of planned behaviour (TPB) to examine how attitudes, subjective norms and perceived control influence self‐management. Unlike prior TPB studies [26, 27], this study adopts a community perspective, exploring the interplay of individual agency and sociocultural context, examining how local factors shape behaviours and considering both internal and external dimensions of norms and control, providing a more nuanced understanding within the Chinese context. This integrated approach will inform targeted policy recommendations to improve self‐management and outcomes.

## 2 Methods

### 2.1 Design

An interpretive qualitative research design was used to explore the mechanisms underlying self‐management behaviours of patients with T2DM in the community. Interpretivism offers an appropriate framework because it focuses on the idea that reality is socially constructed and subjective, and emphasizes understanding the context and meanings individuals or groups attribute to their experiences [28]. This approach aims to understand people's experiences, perspectives, and meanings of a phenomenon in their unique context [29]. In this study, the interpretive approach was most relevant to achieving the study aim and exploring groups of patients with T2DM subjective experiences, perceptions and behavioural motivations, revealing their perspectives on DSM behaviours and their experiences within policy or specific contexts.

The interview data were analyzed using a deductive content analysis approach, utilizing existing theory to focus the research question and predict relationships among variables [28]. Codes, sub‐categories and categories were identified [30] and subsequently structured according to the TPB framework to explain DSM behaviours and to guide our discussion of the findings.

### 2.2 Theoretical Framework

**Figure 1:**
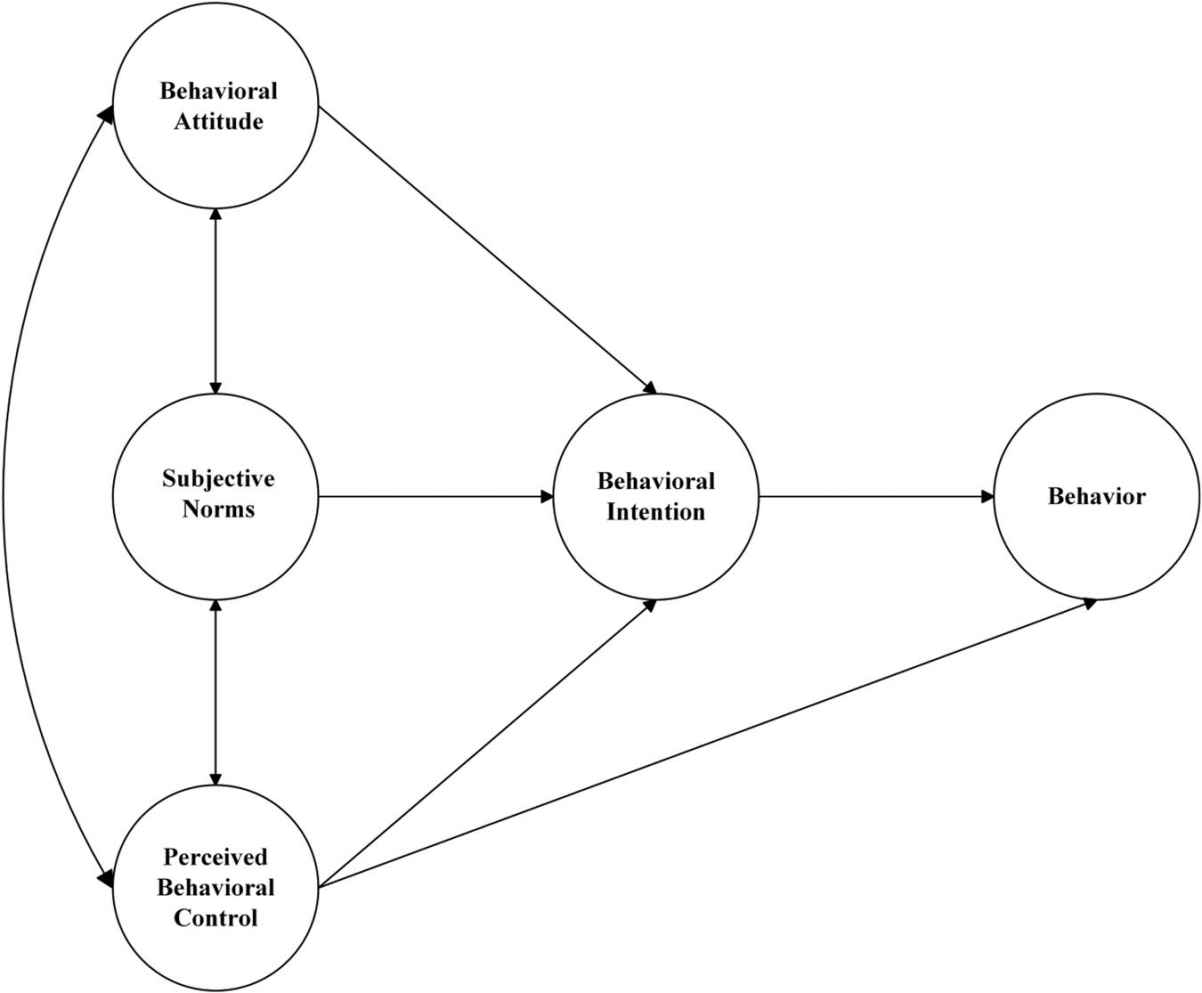
Theory of planned behaviour framework.

The theoretical framework for this study was based on TPB, an important social psychological framework used to predict and explain human behaviour in specific contexts [31]. TPB consists of behavioural attitude (the positive or negative feelings that an individual holds about the behaviour), subjective norms (the extent to which influential individuals or groups influence whether or not an individual adopts a particular behaviour), perceived behavioural control (the individual's past experiences and anticipated obstacles), behavioural intention (the individual's willingness to adopt a particular behaviour) and behaviour (the individual's actual act of taking action) [31], as in Figure 1. TPB suggests that behaviour intention determines behaviour, which in turn is controlled by behaviour attitude, subjective norms and perceived behaviour control, while perceived behaviour control can also directly affect behaviour [32]. TPB has been widely applied in various behavioural domains and has consistently demonstrated its effectiveness in explaining and predicting human behaviour [33]. These constructs align closely with the challenges faced by T2DM. First, self‐management behavioural attitudes refer to the positive or negative attitude of patients with T2DM in the community towards self‐management. Based on perceived benefits and risks, these attitudes influence patients' willingness to adopt self‐management practices. Second, subjective norms of self‐management refer to the perceived social pressure experienced by patients with T2DM to engage in self‐management behaviours, reflecting the impact of social support or cultural stigma on behaviour adoption. Finally, self‐management perceived behavioural control refers to patients with T2DM perceptions of the resources they have and the barriers they encounter, revealing the role of environmental barriers (e.g., healthcare access) and personal resources (e.g., health literacy) in sustaining self‐management. Moreover, our previous research has demonstrated that the TPB theoretical constructs have tremendous predictive ability for self‐management intention and actual self‐management behaviour in patients with T2DM [25]. Given its robust theoretical foundation and empirical support, TPB was selected as the framework for this study.

To develop interview guides, we first conducted a preliminary literature review and held discussions within the project team. The draft was reviewed by experts, and the interview content was revised based on their feedback. The interview questions were designed to align with the framework's structure: behaviour attitude consisted of 2 items: ‘What factors do you think would make individuals with T2DM willing to adhere to DSM behaviours?’ and ‘What factors do you think would make individuals with T2DM reluctant to adhere to DSM behaviours?’ Subjective norm: ‘What groups or individuals do you think influence self‐management behaviours in people with T2DM?’ Perceived behaviour control includes two items: ‘What factors do you think motivate people with T2DM to engage in reasonable self‐management behaviours?’ and ‘What factors do you think prevent people with T2DM from engaging in reasonable self‐management behaviours?’ (see Supporting Information: Additional File 1).

### 2.3 Study Setting and Recruitment

In this study, we employed purposive sampling to recruit participants diagnosed with T2DM who were seeking medical care at a community health service centre in Nanjing, Jiangsu Province, during May 2022. All interviews were conducted before formal data analysis began. However, early analysis of a subset of interviews did inform the focus of later interviews, allowing for a more in‐depth exploration of emerging themes related to the TPB constructs.

The sample size was determined using data saturation [34], whereby participants were continuously recruited until no new information emerged [35].

### 2.4 Inclusion and Exclusion Criteria

Inclusion criteria for participation in the study were: (1) Age ≥ 18 years; (2) Confirmed diagnosis of T2DM, in accordance with the diagnostic criteria for DM introduced by the World Health Organization in 1999; (3) Permanent residents in the community for at least 6 months; (4) Clear consciousness; (5) Good communication and cooperation skills; (6) Informed consent and voluntary participation in this study.

Exclusion Criteria for participation in the study were: (1) Acute diabetes complications (e.g., ketoacidosis and hyperosmolar hyperglycemic state); (3) Suffering from malignant neoplasm or other serious diseases in advanced stages; (4) Comorbid with psychiatric diseases or serious cognitive dysfunction; (5) Severe hearing impairment or speech dysfunction.

### 2.5 Data Collection

Two qualitative researchers with advanced training in public health and nursing, who completed a standardized protocol training programme emphasizing neutral interviewing techniques and bias mitigation strategies. The interviews were organized and hosted by one researcher, while another acted as the recorder, noting key observations and nonverbal cues. Twenty‐one individuals participated in semi‐structured interviews, each with a maximum duration of 60 min. All interviews were audio‐recorded in secure private rooms at the community health centre following informed consent.

Before the interviews, participants were provided with a brief explanation of T2DM self‐management behaviours, which typically include a regular diet and exercise regimen, monitoring of blood glucose levels and medication adherence [36]. To minimize response bias, interviewers used non‐prescriptive language and neutral probes when discussing self‐management concepts. They avoided using value‐laden terms (e.g., ‘correct’ or ‘ideal’) and instead asked open‐ended questions (e.g., ‘Can you describe how you manage your diabetes daily?’). Follow‐up probes (e.g., ‘Can you give an example?’) were used to clarify and validate participants' descriptions. Interviews were conducted individually without family members present to reduce social desirability bias. Participants were assured of confidentiality and encouraged to share their experiences candidly.

These interviews were transcribed verbatim using the ‘Feishu Miaoji’ function in the Feishu V5.26.6 software. Any unrecognized or misrecognized portions were manually entered or modified, resulting in a total of 134,799 words of transcribed data.

### 2.6 Data Analysis

Two‐thirds of the interview samples were selected for initial coding. These 14 interviews were analyzed using NVivo 11.0. A deductive approach was used as an analytical strategy. Theoretical themes and categories driving the analysis were organized around the TPB: behaviour, behavioural attitudes, subjective norms and perceived behavioural control. The codes were duplicated across these 14 samples, but the researchers coded the remaining 7 samples for further assurance and prevention of false saturation. The transcribed interviews were carefully reviewed to identify meaning units, which were then condensed based on their specific content and context. These condensed meaning units were subsequently abstracted, labelled with codes, compared and categorized into sub‐categories and categories (Table 1) [29].

**Table 1:** Data analysis process of self‐management behaviours in community patients with T2DM based on the TPB.

Meaning unit	Condensed meaning unit	Code	Sub‐categories	Category	Theme
I do not measure my blood glucose because I feel like I have it under control.	Having confidence in own blood glucose	Self‐perception	Internal factor	Self‐management behavioural attitude	Self‐management behaviours in patients with T2DM
Sometimes it doesn't matter if I do not take medication for over 10 days.	No discomfort in body without using medicine				
I think it is unnecessary to measure blood sugar.	Lack of attention to blood glucose monitoring	Perceived importance			
Some people do not take diabetes seriously.	Lack of attention to diabetes				
Some people lack the awareness to control their diet.	Lack of attention to dietary control				
A little bit of everything is good for you.	Various foods are beneficial to health.	Understanding of the disease			
Some people believe that injecting insulin can control blood glucose without the need for dietary control	Only taking insulin can control the disease				
The key is to be careful with what you eat.	Just need diet control				
We should talk more about the dangers of diabetes.	Unaware of the dangers of diabetes				
Preventing high blood sugar.	Beneficial for diabetes	Perceived benefits	External factor		
It is for my own health.	Beneficial to the overall body				
The doctor's advice is good for my condition.	Medical advice is beneficial to oneself				
It is good for high blood pressure too.	Beneficial for other chronic diseases				
I want to avoid complications.	Preventing complications				
I once had a stroke after not exercising for over 10 days.	Preventing comorbidities				
If I do not control it, there will be a risk to my life.	Fear of threatening life				
I want to live longer and extend my lifespan.	May prolong life				
This medicine makes me feel better.	Satisfactory medication effects			
I only added this medicine recently because I wasn't managing well.	Unsatisfactory medication effects				
To control my weight.	Weight control				
I stopped taking the medicine because it made my mouth feel sticky and uncomfortable.	Discomfort from side effects of medication	Degree of acceptance			
I am willing to take them if there are no side effects.	Medication without side effects				
When I am a little hungry, it is hard for me to control what I eat.	Diet control easily leads to a feeling of hunger				
I do not feel uncomfortable after eating less.	No discomfort with diet control				
I influence myself.	Control for oneself	Patients themselves	Intrinsic pressure	Subjective norms of self‐management	
My spouse, son and daughter all tell me to control my diet.	Family	Surrounding individuals	Extrinsic pressure		
The doctor advised me to eat small and frequent meals.	Doctor				
I know some people with diabetes, and we talk about medication.	Friend				
The TV programme ‘Road to Health’ and my mobile phone both provide an introduction to diabetes.	Medium	Social groups			
I look at the books they gave me to see what can be eaten and what should be avoided.	Health education organization				
Only the places that sell supplements tell me about precautions.	Healthcare products publicity organization				
I have always liked eating fatty meats.	Eating habits and hobbies	Personal characteristics	Inner factor	Self‐management perceived behavioural control	
Some people are wilful and do not take responsibility for themselves.	Personality factors				
People without health awareness do not care about themselves.	Cultural quality				
Sometimes I do not exercise when I feel tired and uncomfortable.	Physiological experience	Physical and mental experience			
I am scared of needles, especially when being poked by them.	Psychological experience				
Testing blood glucose costs money; I had to do it every day if it didn't.	The cost of blood glucose monitoring	Economic resources			
Some people save by taking one less pill.	The cost of medication				
Those with poor blood sugar control need to be hospitalized.	The cost of hospitalization				
I know someone with a fasting blood sugar of 16, and I recommended soba noodles, but he thought it was too expensive.	The cost of dietary control				
I do not exercise if I am busy at home.	Busy with housework or work	Time management			
Occasionally treats and meals will be indulgent.	Socializing or partying				
If I am bored at home, I will go exercise to keep myself busy.	Ample spare time				
I do not go out in bad weather or rain.	Weather conditions	Environmental suitability	Outer factor		
I like to exercise when the morning air is fresh.	Air quality				
Getting discounted medication requires a blood sugar test.	Chronic Disease Benefit Policy	Policy support			
Now I am on government benefits. I want to live a few more years.	Satisfaction with social welfare				
There used to be a blood sugar testing service nearby, but it stopped coming after COVID‐19.	COVID‐19 affects blood glucose monitoring services				
It is difficult to go out because of my legs, and the places to test my blood sugar are far away.	Distance to blood glucose monitoring service				
I bought a blood glucose metre, but I do not know how to use it.	Lack of blood glucose monitoring techniques	Technical availability			

In the qualitative analysis, the research team systematically codes participants' statements. For instance, the raw quote ‘I avoid checking blood sugar…’ is condensed to ‘Avoidance due to anxiety’, which is then coded as ‘Psychological Barrier’ and falls under the ‘Perceived Behavioural Control’ category of the TPB.

### 2.7 Trustworthiness of Data

The principal investigator spent about a year collecting and analyzing the data, engaging deeply to ensure the credibility of the data. An audit trail was conducted to document the research process to enhance the reliability of the study, including participant recruitment, data collection and data analysis processes. During the interview, the researcher restated information and questioned participants to determine accuracy. Researcher triangulation was conducted by coding the interview transcripts separately by two researchers. Discrepancies were discussed in team meetings. To improve transferability, we described in detail the demographic characteristics of the participants so that readers can judge the applicability of the findings to other contexts.

## 3 Results

### 3.1 Participant Characteristics

The 21 participants (15 female and 6 male) were aged 63–82 years (mean = 71.8 ± 4.72) (Table 2). Most had a primary school education or below (76.2%), and the average duration of T2DM diagnosis was 9.8 years (range: 1–25).

**Table 2:** Basic information of the participants.

Number	Gender	Age (years)	Education level	Duration of diagnosis (years)	Treatment
P1	Female	67	Primary school and below	10	Oral hypoglycaemia agent
P2	Female	69	Primary school and below	12	Oral hypoglycaemia agent
P3	Male	72	Primary school and below	5	Oral hypoglycaemia agent
P4	Male	74	Primary school and below	3	Oral hypoglycaemia agent
P5	Female	73	Senior middle school	5	Oral hypoglycaemia agent
P6	Female	68	Primary school and below	12	Oral hypoglycaemia agent + insulin
P7	Female	75	Junior middle school	10	insulin
P8	Female	69	Primary school and below	23	insulin
P9	Female	75	Primary school and below	1	Oral hypoglycaemia agent
P10	Male	71	Junior middle school	10	Oral hypoglycaemia agent
P11	Male	71	Junior middle school	2	Oral hypoglycaemia agent
P12	Female	82	Primary school and below	25	Oral hypoglycaemia agent
P13	Female	73	Primary school and below	7	Insulin
P14	Female	73	Primary school and below	8	Oral hypoglycaemia agent
P15	Female	67	Primary school and below	18	Oral hypoglycaemia agent
P16	Male	63	Primary school and below	2	Oral hypoglycaemia agent
P17	Male	78	Junior middle school	14	Insulin
P18	Female	79	Primary school and below	15	Oral hypoglycaemia agent
P19	Female	66	Senior middle school	7	Oral hypoglycaemia agent
P20	Female	75	Primary school and below	2	Oral hypoglycaemia agent + insulin
P21	Female	67	Junior middle school	12	Oral hypoglycaemia agent + insulin

**Figure 2:**
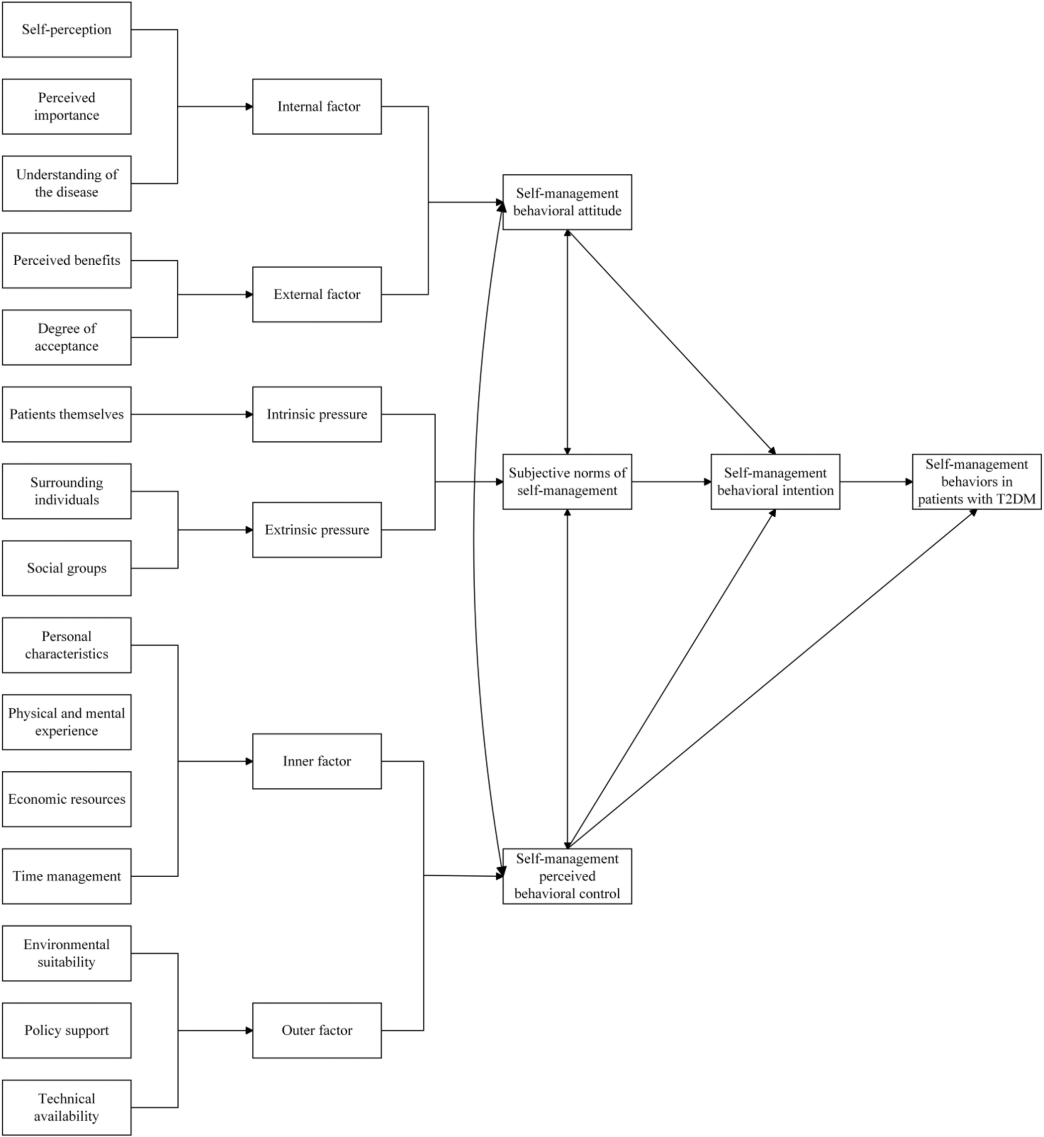
Description of self‐management behaviours of patients with T2DM using the theory of planned behaviour framework.

Figure 2 presented the identified codes, sub‐categories and categories within the TPB framework to explore the mechanisms of self‐management behaviours.

### 3.2 Self‐Management Behavioural Attitude

These attitudes included internal factors formed by patients' subjective perceptions of self‐management, and external factors formed by their objective experiences with these behaviours. Internal factors included participants' self‐perceptions, the perceived importance of self‐management and their understanding of the disease. External factors included the perceived benefits and acceptance of self‐management behaviours.

### 3.3 Internal Factor

Patients with positive self‐perception exhibited negative attitudes towards self‐management behaviours. The patients emphasized their subjective feelings or the easing of their symptoms. They believed that if they did not experience obvious discomfort, there was no need to monitor blood glucose levels or take medication.

I don't measure my blood glucose because I feel like I've got it under control.

(P20)

Sometimes it doesn't matter if I don't take medication for over 10 days.

(P3)

Patients who perceived self‐management behaviours or their disease as unimportant exhibited negative attitudes. They considered these self‐management behaviours unnecessary.

I think it's unnecessary to measure blood sugar.

(P13)

Some people lack the awareness to control their diet.

(P11)

Some patients lacked sufficient understanding of their disease, leading to a negative attitude towards self‐management. They believed that the disease could be controlled solely through dietary management or insulin use.

Some people believe that injecting insulin can control blood glucose without the need for dietary control.

(P6)

The key is to be careful with what you eat.

(P18)

### 3.4 External Factor

Some patients who perceived benefits from self‐management behaviours or found these practices manageable and comfortable exhibited more positive attitudes.

This medicine makes me feel better.

(P4)

I once had a stroke after not exercising for over ten days.

(P6)

In addition, patients vary in their acceptance of self‐management behaviours. Some patients exhibited negative attitudes due to the intolerable side effects and discomfort caused by medications, as well as the hunger resulting from dietary restrictions.

I stopped taking the medicine because it made my mouth feel sticky and uncomfortable.

(P3)

I am willing to take them if there are no side effects.

(P4)

When I'm a little hungry, it's hard for me to control what I eat.

(P8)

### 3.5 Subjective Norms of Self‐Management

Subjective norms of self‐management included intrinsic pressure from the patients themselves as well as extrinsic pressure from the surrounding individuals and social groups.

### 3.6 Intrinsic Pressure

Some patients' self‐management behaviours were influenced by themselves and driven by internal motivations.

I influence myself.

(P8)

### 3.7 Extrinsic Pressure

Encouragement and reminders from surrounding individuals prompted patients to engage in self‐management behaviours. Patients trusted and followed the suggestions of family members, doctors or friends, and most patients mentioned the influence of family members on their self‐management behaviours.

My spouse, son, and daughter all tell me to control my diet.

(P20)

The doctor advised me to eat small and frequent meals.

(P12)

I know some people with diabetes, and we talk about medication.

(P12)

Additionally, broader social groups, such as media, health education organizations and healthcare promotion organizations, also contributed to the perceived social pressure to engage in self‐management.

I look at the books they gave me to see what can be eaten and what should be avoided.

(P2)

Only the places that sell supplements tell me about precautions.

(P8)

The TV program ‘Road to Health’ and my mobile phone both provide an introduction to diabetes.

(P17)

### 3.8 Self‐Management Perceived Behavioural Control

Self‐management perceived behavioural control consisted of inner and outer factors. The inner factors included personal characteristics, physical and mental experience, economic resources and time management. The outer factors included environmental suitability, policy support and technical availability.

### 3.9 Inner Factor

Personal characteristics such as eating habits, personality traits and cultural quality were the influencing factors of self‐management behaviours. Multiple patient accounts indicated that long‐established dietary preferences were often difficult to alter and directly hindered their ability to control their diet. Furthermore, some patients exhibited reduced willingness to consistently engage in self‐management behaviours due to personality traits lacking in responsibility and self‐discipline. Additionally, weak health awareness limited patients' emphasis on self‐management behaviours.

I've always liked eating fatty meats.

(P8)

Some people are willful and don't take responsibility for themselves.

(P11)

People without health awareness don't care about themselves.

(P11)

This study found that physical and mental experience affected patients' self‐management behaviours. Regarding physical experiences, some patients discontinued exercise due to bodily fatigue. Regarding psychological experiences, some patients experienced anticipatory anxiety about potential pain during insulin injections or blood glucose monitoring. This fear or nervousness similarly negatively influenced their self‐management behaviours.

Sometimes I don't exercise when I feel tired and uncomfortable.

(P19)

I'm scared of needles, especially when being poked by them.

(P12)

In addition, low economic affordability hindered patients' self‐management. Economic pressures limited patients' blood glucose monitoring and adherence to medication, and also affected their willingness to adjust their diets.

Testing blood glucose costs money; I'd do it every day if it didn't.

(P8)

I know someone with a fasting blood sugar of 16, and I recommended soba noodles, but he thought it was too expensive.

(P4)

Poor time management also posed a challenge. Some patients reported that they did not exercise when busy with work and indulged in their diet during parties.

I don't exercise if I'm busy at home.

(P12)

Occasionally treats and meals will be indulgent.

(P11)

### 3.10 Outer Factor

Environmental suitability influenced patients' physical activity. Patients indicated they were more willing to engage in outdoor activities when the air was fresh; conversely, they tended to avoid going out in unfavourable weather conditions.

I like to exercise when the morning air is fresh.

(P4)

Supportive policies promoted patients' self‐management behaviours. Patients reported needing to test their blood glucose before getting discounted medications, which strengthened their self‐management behaviours. Social welfare benefits also enhanced patients' motivation for self‐management.

Getting discounted medication requires a blood sugar test.

(P12)

Now I'm on government benefits. I want to live a few more years.

(P13)

Some patients purchased blood glucose metres but did not know how to use them. The lack of blood glucose monitoring techniques hindered their self‐management behaviours.

I bought a blood glucose meter but I don't know how to use it.

(P8)

## 4 Discussion

In this study, we conducted interviews with 21 participants to investigate the mechanisms underlying self‐management behaviours of patients with T2DM in the community, utilizing the TPB as the guiding framework. The findings reveal that factors affecting the self‐management behaviours of patients with T2DM in the community include their behavioural attitudes, subjective norms and perceived behavioural control. Based on our findings regarding patients' attitudes, subjective norms and perceived behavioural control, the policy recommendations derived from this study offer actionable strategies for both patients and healthcare providers to improve self‐management outcomes.

### 4.1 Self‐Management Behavioural Attitude

Behavioural attitudes directly influence patients' willingness to engage in self‐management. Our results suggest that individuals who perceive the benefits of self‐management actions without associated discomfort are more likely to adopt favourable attitudes towards these actions. This aligns with previous literature [37], which indicated that individuals' lack of awareness of benefits often leads to negative perceptions. For instance, certain patients may monitor their blood sugar levels to assess their health status. However, when these levels are within a normal range, they may experience relief and refrain from further self‐management activities. Some patients avoid self‐management practices because of the stress associated with elevated blood glucose levels. Positive self‐management attitudes can be more effectively fostered when patients experience tangible benefits from effective self‐management practices. These preliminary findings suggest the need to enhance health awareness and promote self‐management to optimize disease prevention and treatment. This aligns with other research [38], highlighting the critical need for effective follow‐up for patients with T2DM and timely patient–provider communication.

### 4.2 Subjective Norms of Self‐Management

Our research observed that participants reported engaging in self‐management practices when experiencing pressure from various sources. This pressure can be intrinsic pressure, potentially associated with the patient's role itself, which encompasses the social status, rights and obligations imposed by society on individuals with illness [9]. Consistent with previous research [39], we observed that participants who accepted the patient's role were more inclined to engage in self‐management. From the perspective of external pressures, individuals with type 2 diabetes may experience certain external pressures from surrounding groups represented by family members (including partners, children and relatives), doctors, friends (including neighbours and fellow diabetes patients), as well as societal groups such as media outlets, health education organizations and health supplement promotion groups, can exert external pressures on individuals with type 2 diabetes. Such pressures compel patients to fully adhere to diabetes management protocols, thereby motivating them to engage in self‐management behaviours.

At the same time, these groups may also provide social support to patients. Evidence indicates that increased social support contributes to improved self‐management among individuals with type 2 diabetes [40, 41]. Social support can be divided into formal and informal support. Formal social support, an essential factor in facilitating self‐management, is primarily provided by official organizations at various governmental levels, including institutions and corporations, which offer assistance through social security systems, healthcare services and policies targeted at aiding and respecting the elderly [42]. In contrast, informal social support primarily stems from family members, neighbours, friends, peer groups and others, who offer emotional, behavioural and informational assistance [43].

Previous investigations into formal social support for individuals with T2DM have predominantly focused on government organizations. However, our study reveals an additional role of community healthcare organization, which increasingly contribute to the self‐management of patients with T2DM. These organizations are playing an increasingly important role in enhancing effective self‐management behaviours of this patient population.

### 4.3 Self‐Management Perceived Behavioural Control

The observed factors were explored within the TPB framework, with inner and outer elements reflecting participants' descriptions of facilitators and barriers influencing their perceived control over self‐management participation and behaviour. In terms of inner factors, our study aligns with prior research [44], showing the effect of personality traits on blood glucose levels in T2DM. Rigorous individuals display superior self‐management [45] due to stronger social support networks. In contrast, Li et al. studies [46] indicate that extroverts exhibit weaker self‐management skills, possibly due to the complexity of hospitalized patients studied. Consistent with earlier findings [47], higher‐educated patients exhibit great self‐management or perceived behavioural control, possibly owing to positive health beliefs and proactive information seeking.

Physical discomfort and psychological experiences, particularly anxiety associated with blood glucose monitoring [12, 48, 49], also pose barriers to self‐management in people with T2DM. This may be related to age and duration. Older age and longer disease duration can reduce patients' sense of control, as functional decline, multiple chronic conditions and diabetes distress may hinder effective self‐management. However, time and cost constraints hinder self‐management practices, especially for those with limited financial resources for medication, diet and blood sugar monitoring [50].

Concerning the outer factors, self‐management behaviours are driven primarily by high environmental suitability, robust policy support and broad technology availability, consistent with the results of earlier studies [51, 52]. This conclusion can be attributed to several main factors. First, individuals are more inclined to adopt self‐management practices in an enabling environment. Second, supportive policies that improve well‐being and reduce financial burden and increased availability of technology, all contribute to greater engagement in and reinforcement of self‐management behaviours.

### 4.4 Policy Implications

Based on the findings of this study, the following policy recommendations are proposed to further improve self‐management behaviours among community‐dwelling individuals with type 2 diabetes, enhance their quality of life and reduce the disease burden on families and society, presented in Table 3. These recommendations address the need for encouraging family members to support the DSM, strengthening follow‐up procedures, guiding on the patient role more clearly, improving personal coping strategies, promoting social support system and broadening the scope of policy protection. Additionally, the policy suggestions aim to empower individuals with T2DM to actively manage their condition, thereby reducing the overall impact of the condition. These suggestions also seek to enhance individual health, optimize healthcare resources utilization, create a more sustainable healthcare system and better prepare for the increasing prevalence of this condition.

**Table 3:** Policy recommendations for self‐management behaviours of people with T2DM in the community.

Policy recommendations	Specific measures	
Self‐management behavioural attitude	Encourage family members to support the DSM	Conduct structured health education for family members to help them understand key aspects of T2DM management, enabling them to better support patients. Train family members in daily motivational techniques to provide ongoing emotional support and behavioural reinforcement for patients.
	Strengthen follow‐up procedures [53]	In family doctor contract services, patients are encouraged to involve family members in follow‐up plans. Strengthen training for primary care providers in communication skills with patients and their families to enhance the relevance and approachability of follow‐up services.
Subjective norms of self‐management	Guide the patient role more clearly	Establishing a good doctor‐patient relationship and guide psychological changes at the right time. Utilize community bulletin boards, health lectures and other channels to promote successful patient self‐management case studies, leveraging their role as role models.
	Promote social support system [54]	Encourage family members to accompany the doctor and participate in health education. Facilitate peer support groups and community‐based activities for shared learning and emotional support
Self‐management perceived behavioural control	Improve personal coping strategies [55]	Provide patients with targeted self‐management skills training, such as simple meal planning and low‐intensity exercise methods. Strengthen labour rights protections for working patients to ensure they have sufficient time for disease management.
	Broaden the scope of policy protection	Increase the number of indoor public places for physical activities and strengthen air treatment; Establish additional convenient, free blood glucose monitoring stations in communities, providing simple operational guidance; Optimize chronic disease medication policies to ensure drug efficacy and accessibility while prioritizing side effect management.

### 4.5 Limitations

While our findings offer valuable insights, it is important to recognize the limitations of our study. First, the research was conducted only among type 2 diabetes patients from a single community health service centre, which may limit the generalizability of the results. Future studies could include multiple regions and community health service centres of varying sizes, allowing for more in‐depth and comparative analyses. Second, this study focused exclusively on the patient perspective and did not incorporate views from other stakeholders. Future studies should incorporate the views of family members and healthcare providers to capture a more comprehensive understanding of the barriers and facilitators to DSM. Moreover, Future research could conduct separate interviews with patients who successfully implemented DSM and those who did not, thereby revealing the key factors underlying these behavioural differences. Finally, the predominance of older female participants reflects the higher prevalence of T2DM in this group but may also introduce selection bias. Future work should aim for a more diverse and balanced sample to enhance representativeness.

## 5 Conclusion

Guided by the TPB, this study examines the mechanisms influencing self‐management behaviours among patients with type 2 diabetes. The analysis identifies and organizes the contributing factors into three dimensions: attitudes towards self‐management, subjective norms and perceived behavioural control. The TPB framework was well‐suited for capturing the key psychosocial determinants shaping patients' intentions and self‐management practices. Based on these findings, we propose policy recommendations to encourage family members to support the DSM, foster social support and establish a supportive community environment that provides accessible and reliable diabetes‐related information.

## Author Contributions

**Jinxin Li:** conceptualization, investigation, methodology, writing – original draft preparation, writing – review and editing, formal analysis, data curation. **Yue Zhang:** conceptualization, investigation, methodology, writing – original draft preparation, writing – review and editing, formal analysis, data curation. **Nan Zhao:** methodology, formal analysis, data curation. **Jianxiao Zheng:** formal analysis, data curation. **Yang Wang:** visualization, writing – review and editing. **Jiajia Dong:** visualization, writing – review and editing. **Jianwen Zhu:** methodology, writing – review and editing. **Jie Fu:** writing – review and editing. **Hong Fan:** conceptualization, methodology, supervision, writing – review and editing.

## Ethics Statement

This study strictly adhered to the ethical principles of the Declaration of Helsinki. The study protocol was approved by the ethics committee of Nanjing Medical University (approval no. 941).

## Consent

Participant confidentiality was rigorously protected, and informed consent was obtained from all individual participants included in the study.

## Conflicts of Interest

The authors declare no conflicts of interest.

## Supporting information


**Table S1:** Interviewee Personal Information Form. **Table S2:** Interview Outline.

## Data Availability

To protect the confidentiality of the respondents, data will not be shared, but are available from the corresponding author on reasonable request.
