# Supplementary material for: A Qualitative Study Exploring the Mechanisms Underlying Self‐Management Behaviours Among Community‐Dwelling Type 2 Diabetes Mellitus Patients in Nanjing, China
Source: Health Expect. 2025 Dec 8;28(6):e70522. doi: 10.1111/hex.70522 (PMC12683677; doi:10.1111/hex.70522)
Supplement: Supplementary file 1 — Table S1: Interviewee Personal Information Form. Table S2: Interview Outline. [file HEX-28-e70522-s001.pdf]

Interview materials

Table S1 Interviewee Personal Information Form

NO.☐

Name		Gender		Age (year)	
Duration of Diagnosis (year)		Education Level		Treatment Method	
Telephone Number		Other Contact Information			

Do you accept interview recording: ☐Yes ☐No

Do you need the interview recording file: ☐Yes ☐No

Interview Date: [Month Day, Year]

Time: [Start Hour:Minute AM/PM] – [End Hour:Minute AM/PM]

Table S2 Interview Outline

Dimension	Item
Behavior attitude	What factors do you think would make individuals with T2DM willing to adhere to diabetes self-management behaviors?
	Dietary control
	Regular exercise
	Medication adherence
	Blood glucose monitoring
	What factors do you think would make individuals with T2DM reluctant to adhere to diabetes self-management behaviors?
	Dietary control
	Regular exercise
	Medication adherence
	Blood glucose monitoring
Subjective norm	What groups or individuals do you think influence self-management behaviors in people with T2DM?
	Dietary control
	Regular exercise
	Medication adherence
	Blood glucose monitoring
Perceived behavior control	What factors do you think motivate people with T2DM to engage in reasonable self-management behaviors?
	Dietary control
	Regular exercise
	Medication adherence
	Blood glucose monitoring
	What factors do you think prevent people with T2DM from engaging in reasonable self-management behaviors?
	Dietary control
	Regular exercise

	Medication adherence
	Blood glucose monitoring
